# Recurrent hormone-binding domain truncated *ESR1* amplifications in primary endometrial cancers suggest their implication in hormone independent growth

**DOI:** 10.1038/srep25521

**Published:** 2016-05-10

**Authors:** Frederik Holst, Erling A. Hoivik, William J. Gibson, Amaro Taylor-Weiner, Steven E. Schumacher, Yan W. Asmann, Patrick Grossmann, Jone Trovik, Brian M. Necela, E. Aubrey Thompson, Matthew Meyerson, Rameen Beroukhim, Helga B. Salvesen, Andrew D. Cherniack

**Affiliations:** 1 Centre for Cancer Biomarkers, Department of Clinical Science, The University of Bergen, Norway; 2 KG Jebsen Center for Precision Medicine in Gynecologic Cancer, Department of Gynecology and Obstetrics, Haukeland University Hospital Bergen, Norway; 3 Department of Cancer Biology, Dana-Farber Cancer Institute, Boston, Massachusetts, USA; 4 Broad Institute of Harvard and MIT, Cambridge, Massachusetts, USA; 5 Department of Medical Oncology, Dana-Farber Cancer Institute, Boston, Massachusetts, USA; 6 Department of Medicine, Brigham and Women’s Hospital, Harvard Medical School, Boston, Massachusetts, USA; 7 Department of Pathology, Brigham and Women’s Hospital, Boston, Massachusetts 02115, USA; 8 Department of Health Sciences Research, Mayo Clinic Cancer Center, Jacksonville, Florida 32224, USA; 9 Department of Radiation Oncology, Dana-Farber Cancer Institute, Brigham and Women’s Hospital, Harvard Medical School, Boston, Massachusetts, USA; 10 Department of Biostatistics & Computational Biology, Dana-Farber Cancer Institute, Boston, MA, USA; 11 Department of Cancer Biology, Mayo Clinic Cancer Center, Jacksonville, Florida.

## Abstract

The estrogen receptor alpha (ERα) is highly expressed in both endometrial and breast cancers, and represents the most prevalent therapeutic target in breast cancer. However, anti-estrogen therapy has not been shown to be effective in endometrial cancer. Recently it has been shown that hormone-binding domain alterations of ERα in breast cancer contribute to acquired resistance to anti-estrogen therapy. In analyses of genomic data from The Cancer Genome Atlas (TCGA), we observe that endometrial carcinomas manifest recurrent *ESR1* gene amplifications that truncate the hormone-binding domain encoding region of *ESR1* and are associated with reduced mRNA expression of exons encoding the hormone-binding domain. These findings support a role for hormone-binding alterations of ERα in primary endometrial cancer, with potentially important therapeutic implications.

Endometrial cancer (EC) is the fourth most common malignancy of women and the most common pelvic gynecological malignancy in countries with advanced industrialization^1^,^2^. But approved targeted therapies are still not in use today^3^,^4^. ERα, encoded by the gene *ESR1*, is known to be an important driver of cell proliferation^5^ and has been identified as a risk locus in breast cancer^6^,^7^. Both breast as well as endometrial cancer are estrogen dependent and express the estrogen receptor alpha (ERα) to a similar extent^8^,^9^,^10^,^11^.

While ERα constitutes the most frequently inhibited therapeutic target in breast cancer^9^, anti-estrogen therapy has shown inconsistent results and mostly a very limited effect in endometrial cancers^12^,^13^,^14^,^15^,^16^,^17^,^18^. The estrogen antagonist Tamoxifen can even increase the risk of carcinogenesis^19^,^20^,^21^. Consequently anti-estrogen therapy does not constitute a component of standard therapy of EC^3^,^4^. Since mutations and alternative splicing of *ESR1* that alter the hormone-binding domain have been shown to generate hormone independence or resistance to anti-estrogen therapy in breast and endometrial cancers^22^,^23^,^24^,^25^,^26^,^27^,^28^,^29^,^30^,^31^, related genetic alterations could play a role for therapy outcome in primary endometrial carcinoma.

Recent studies identified mutations of *ESR1* in breast cancer that alter their hormone binding domain coding sequence, to be linked to endocrine therapy resistance in a metastatic setting^26^,^27^,^28^. One study by Li *et al*. even demonstrates an *ESR1* fusion in endocrine treatment resistant breast cancer, truncating the hormone-binding domain coding exons^28^, while a later study by Veeraraghavan *et al*. identified evidence for another type of recurrent ERα-altering gene fusions in this tumor type^32^. However, structural genetic alterations of *ESR1* have not been suggested to play a role in endometrial cancer carcinogenesis. Due to the potential importance of such *ESR1* alterations in endometrial cancer, we analyzed an tumor test subset of 29 primary endometrial cancers for somatic gene copy-number alterations (SCNA) and explored The Cancer Genome Atlas (TCGA)^33^ for concerning SCNA and mRNA expression data of endometrial carcinoma.

## Results

**Figure 1:**
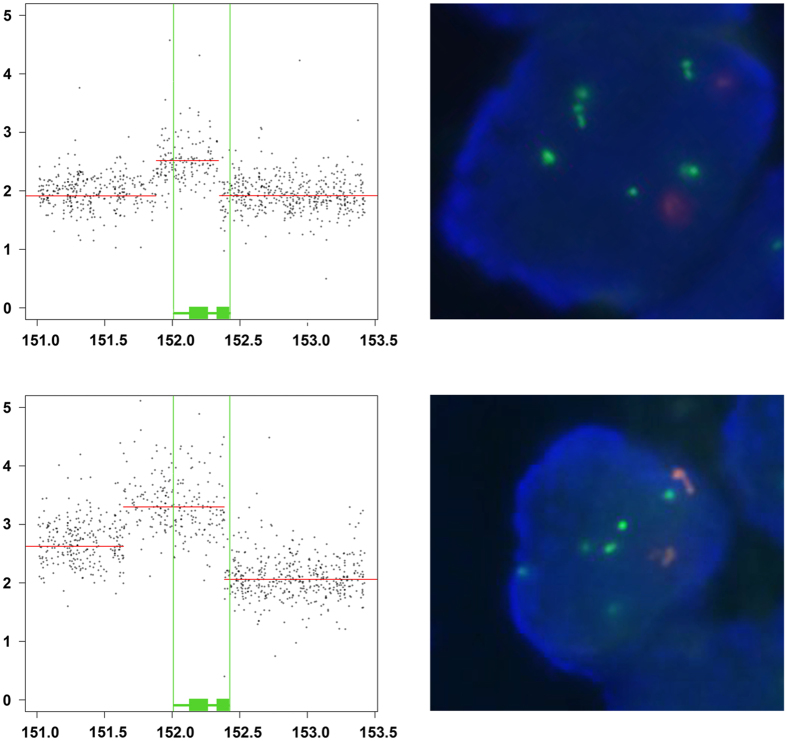
Truncated ESR1 amplifications in two metastatic endometrial carcinomas. Dot plots of *ESR1* copy-numbers (y-axis) determined by GeneChip measurements (grey dots) of two metastatic endometrial carcinomas (above: #4, below #2) are shown on the left. Horizontal red lines indicate the segmented copy-number level of chromosomal positions (mega base pairs) on chromosome 6 (x-axis). Position of full length *ESR1* (vertical green lines) as well as ESR1 exons 1-4 and 5-8 are indicated as green rectangles (see also Figure 2). Regarding FISH signals of ESR1 (green) and centromere 6 (orange) within a tumor nucleus (blue) are shown on the right. FISH and regarding GeneChip copy-number data of 28 metastatic endometrial carcinoma are summarized in Appendix A. FISH analyses of these tumors are documented in Supplementary Optical Dataset S1.

Across a cancer study subset of 29 primary endometrial carcinomas that had gone on to metastasize, we characterized the copy-number changes by GeneChips and validated amplifications of *ESR1* in these cancers by fluorescence *in-situ* hybridization (FISH). The Pearson correlation of *ESR1* GeneChip copy numbers with FISH determined absolute average *ESR1* copy numbers per nucleus and average *ESR1* to centromere 6 (CEN6) ratios were r = 0.743 (p < 0.001) and r = 0.774 (p < 0.001) respectively (Appendix A, Fig. 1, Supplementary Figures S1 and S2, Supplementary Optical Dataset S1).

Four of these tumors exhibited focal *ESR1* amplification determined by GeneChips, of which two amplifications showed 3′ truncations of *ESR1* (Δ exon 6–8 or 7–8) that would remove the hormone-binding domain (Appendix A, Fig. 1). We therefore explored the prevalence of *ESR1*-truncating amplifications across uterine corpus endometrial carcinoma within The Cancer Genome Atlas (TCGA)^33^.

### Hormone-binding domain truncated *ESR1* amplifications in primary endometrial cancers

In the TCGA data subset of 539 endometrial carcinomas analyzed, we identified 88 (16.3%) cases with amplifications encompassing or overlapping *ESR1*. 46.6% of these were histologically defined serous and 75.0% of the tumors with *ESR1* amplification were clustered within the serous like copy-number high molecular subtype according to TCGA^34^. The *ESR1* amplifications were focal (less than half a chromosome arm in length) in 36 cases (6.7%) of tumors, and had a significantly higher rate of amplification than the genome-wide average (q = 5.75 × 10^−4^). Mapping of the overlap between amplifications across tumors identified *ESR1* only as the most likely gene target (see methods).

**Figure 2:**
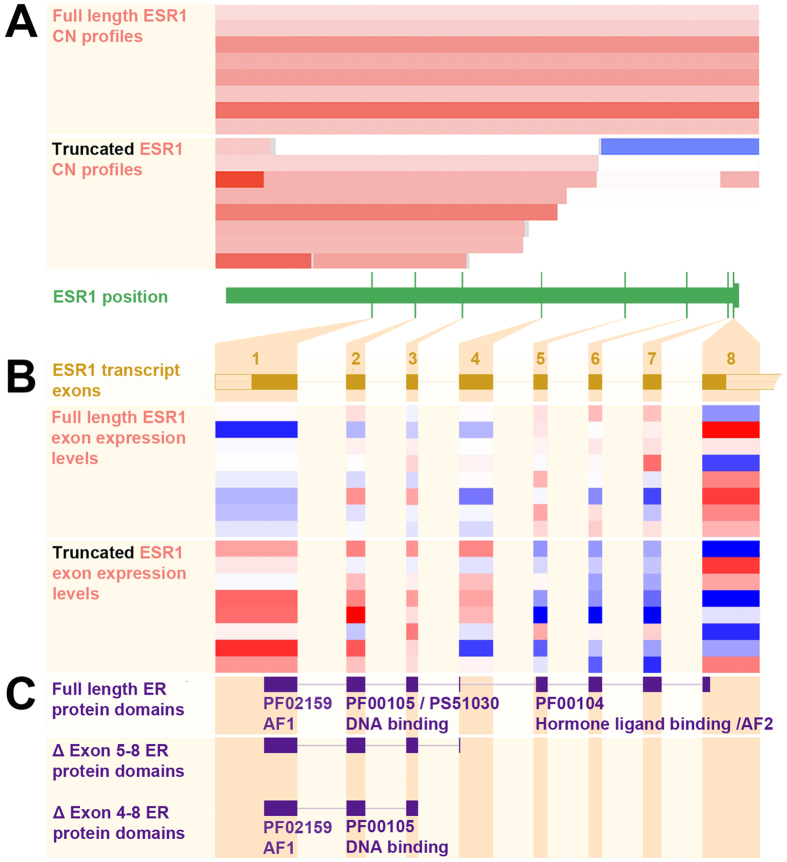
Truncated *ESR1* amplifications in TCGA endometrial carcinomas. Log2 *ESR1* copy number ratios of eight uterine corpus endometrial carcinomas with *ESR1* full-length amplification and eight carcinomas with *ESR1* truncating copy-number alterations are shown in horizontal bars (increased: red, normal/neutral: white, decreased: blue) (A). The corresponding heatmap of exon expression is estimated from RNA-Seq data (normalized relative higher: red, neutral: white, lower: blue) (B). Corresponding ER protein domains according to PROSITE (PS) and Pfam (PF) databases (http://www.ebi.ac.uk/interpro/) are shown in panel C (see http://www.ensembl.orgfor ESR1 transcript variants).

These amplifications appeared to truncate the hormone-binding domain encoding region in seven cases (1.3% of the entire dataset; and 19.4% of cases with focal *ESR1* amplification) and to retain exons 1–4 or 1–3, encoding the n-terminal *ESR1* transactivation domain (AF1) and DNA-binding domains. Another case without *ESR1* amplification exhibited a heterozygous deletion of exons encoding the hormone-binding domain (Fig. 2), for a total apparent *ESR1* truncation rate of 1.5% over all tumors. In one additional TCGA case, we detected a hormone-binding domain (exons 4–8) truncating *ESR1*-*SYNE1* mRNA fusion (Appendix B). Eight of these nine tumors were molecularly classified as being in the serous like copy-number high subgroup (4.3% of this subgroup)^35^.

### Association of *ESR1* exon copy numbers with mRNA expression

The *ESR1* truncation events are associated with decreased mRNA expression of the truncated exons encoding the hormone-binding domain (exons 5–8) compared to the transactivation and DNA-binding domains (exons 1–4) (p < 0.001) (Fig. 2 and Appendix C). We compared the normalized *ESR1* expression values estimated from RNA-Seq data for the eight tumors exhibiting amplified, truncated *ESR1* to those from eight tumors selected on the basis of exhibiting similarly focal *ESR1* amplifications that lack intragenic breakpoints. The average ratio between expression levels of exons 1–4 and 5–8 is 2.1-fold higher among truncated tumors relative to these controls (p = 0.003). We also confirmed this relation after replacing the eight *ESR1*-amplified controls with all 545 tumors profiled by TCGA. In this comparison, the ratio of expression levels between exons 1–4 and 5–8 is 2.2-fold higher in *ESR1*-truncated tumors (p < 0.001).

In contrast, TCGA breast cancers exhibit *ESR1* truncations on DNA-level less than half as often (7 of 1080; 0.65%) as observed in endometrial cancer and had increased expression of exons 1–2, but not of the full DNA-binding domain (Appendix D). These data suggest that the amplified truncations and associated mRNA profiles we describe in endometrial cancer are not frequent in breast cancer.

## Discussion

The gene truncations we report in endometrial carcinoma disrupt the hormone-binding domain encoding sequence of *ESR1*. Similarly, mRNA splice variants lacking one or more of exons 5–8, encoding the hormone-binding domain, have been described in normal^35^,^36^,^37^,^38^ and malignant^22^,^23^,^35^,^36^,^37^,^39^ breast as well as in normal^22^,^40^,^41^,^42^,^43^,^44^,^45^ and malignant^42^,^43^,^44^,^45^,^46^ endometrial tissue. Point mutations of the ligand binding domain encoding sequence of *ESR1* have also been described to occur in both breast and endometrial cancers^25^,^26^,^27^,^30^,^47^,^48^.

Both splice variants and point mutations involving the *ESR1* hormone-binding domain have been associated with hormone-independent ERα activity. The point mutations found in both breast and endometrial cancers have been shown to enable ligand-binding independent transcriptional activity^26^,^30^,^48^,^49^,^50^ and have been related to acquired resistance to anti-estrogen therapy in breast cancer^26^,^27^,^28^. Excisions of exons 5 and 7 by alternative splicing have also been shown to constitutively activate ERα^22^,^23^,^30^ and have been associated with hormone independent growth in both breast and endometrial cancer^22^,^23^,^24^,^31^. These findings raise the hypothesis that the *ESR1* truncations we report may also generate hormone-independent ERα activity.

In breast cancer, point mutations in the ligand-binding domain occur in 20–50% of tumors that have acquired resistance to anti-estrogen therapy^26^,^27^ but only in 0.2% of primary cancers^51^. In endometrial cancer, however, point mutations and in-frame deletions altering the ligand binding domain occur in 2.8% of primary endometrial cancers^26^,^51^. Similarly the recurrent *ESR1* truncations we report appear to be much more frequent in primary endometrial carcinoma than in primary breast cancers.

Anti-estrogen therapy with estrogen antagonists or aromatase inhibitors is standard first-line treatment for ERα-positive breast cancers, but has been associated with only a low rate (~10%) of overall response among endometrial cancers^13^,^16^,^17^,^18^ and is not a standard treatment for endometrial cancer^3^,^4^. In some cases, anti-estrogens such as Tamoxifen can even induce proliferation effect on endometrial cancer cells^52^,^53^ and normal endometrial tissue^54^ and increase the risk of endometrial carcinogenesis^19^,^20^,^21^. Splice variants of *ESR1* that alter the hormone-binding domain have been associated with ERα activation by Tamoxifen in endometrial cancer cells^24^. The effect of estrogen antagonists on ERα encoded by the truncated forms of *ESR1* that we have detected should also be tested, and all alterations of the *ESR1* ligand-binding domain should be evaluated as potential biomarkers of anti-estrogen therapy resistance. Conversely, the absence of such alterations should be evaluated as a biomarker of anti-estrogen sensitivity, potentially opening up a new therapeutic option for a subset of patients with endometrial cancer.

## Methods

### GeneChip analysis

For our study subset of 29 primary endometrial tumors, gene copy-number data were determined by Affymetrix SNP 6.0 microarray analysis as described earlier^55^. GeneChip probe intensities are normalized across samples and circular binary segmentation is performed. Areas harboring germline CNVs are removed from the final segmented copy-number output. The range of birdseed call rates in this cohort was 92.6–99.3% with an average call rate of 97.1%. For TCGA copy-number data, level 3 segmented log2 copy-number data were used in analysis. For both datasets, log2 copy-number values are calculated as ratios relative to the genome wide average according to standard procedures^56^,^57^,^58^,^59^. These gene copy-number data were visualized using the IGV viewer software^60^. Linear gene level copy-number data were derived by GISTIC^55^,^59^. All TCGA DNA copy-number data (2015-06-01 stddata 2015-04-02 regular peel-off) can be accessed through the TCGA Copy Number Portal^57^.

### RNA-Seq analysis

Reads per kilobase per million (RPKM)^61^ RNA exon expression quantification values were normalized and RPKM 0 was assigned 0.1 (Appendices C+D). Exons were compared using inverted log2 of normalized values. A two tailed Mann-Whitney-U-Test was applied to test for statistical significance of differences. P-values < 0.05 were considered statistically significant. Paired-end RNA-seq fusion transcript analysis of TCGA RNA-sequencing data from 295 tumors to detect mRNA fusions was performed using SnowShoes-FTD as described earlier^62^,^63^,^64^. Parameters used to define a fusion transcript of high confidence were at least two unique fusion junction spanning split reads within the dataset and at least five encompassing reads^65^. RNA-Seq data were taken from the TCGA database http://cancergenome.nih.gov

### FISH analysis

FISH was performed without RNase treatment as described earlier^66^. Pearson correlation coefficients and regarding p-values (two sided t-test) were generated using SPSS (Statistical Package of Social Science) version 20.0.0 applying standard bootstrapping. P-values < 0.05 were considered statistically significant.

### Tumor samples and DNA extraction

This study has been approved by the Norwegian Data Inspectorate (961478-2), the Norwegian Social Science Data Services (15501) and the local Institutional Review Board (REKIII nr. 052.01) and the BROAD institute, MA, USA and methods were carried out in accordance with these approved guidelines. The 29 metastatic high grade primary tumor samples were obtained with documented informed consent in a patient based setting (Sept 2002-Sept 2012) from the Department of Obstetrics and Gynaecology, Section of Gynaecological Cancer, Haukeland University Hospital, Bergen, Norway. Biopsies were snap frozen in nitrogen and stored at minus 80 °C until DNA extraction. Tumor purity was assessed based on histology sections obtained by microtome prior to DNA extraction. DNA extraction was performed using samples with estimated tumor purity ≥50% as previously described^7^.

## Additional Information

**How to cite this article**: Holst, F. *et al*. Recurrent hormone-binding domain truncated *ESR1* amplifications in primary endometrial cancers suggest their implication in hormone independent growth. *Sci. Rep.* **6**, 25521; doi: 10.1038/srep25521 (2016).

## Supplementary Material

Supplementary Information

Supplementary Information
