# Corrigendum: Recurrent hormone-binding domain truncated *ESR1* amplifications in primary endometrial cancers suggest their implication in hormone independent growth

**DOI:** 10.1038/srep27960

**Published:** 2016-06-24

**Authors:** Frederik Holst, Erling A. Hoivik, William J. Gibson, Amaro Taylor-Weiner, Steven E. Schumacher, Yan W. Asmann, Patrick Grossmann, Jone Trovik, Brian M. Necela, E. Aubrey Thompson, Matthew Meyerson, Rameen Beroukhim, Helga B. Salvesen, Andrew D. Cherniack

Scientific Reports 6: Article number: 2552110.1038/srep25521; published online: 05102016; updated: 06242016

In the original version of this Article, there were errors in Affiliation 2 which was incorrectly given as ‘KG Jebsen Center for Precision Medicine in Gynecologic Cancer, Department of Gynecology and Obstetrics, Haukeland University Hospital Bergen, Norway’. The correct affiliation is listed below:

‘Department of Gynecology and Obstetrics, Haukeland University Hospital Bergen, Norway’.

This error has now been corrected in the PDF and HTML versions of the Article.

