# Corrigendum: Recurrent hormone-binding domain truncated *ESR1* amplifications in primary endometrial cancers suggest their implication in hormone independent growth

**DOI:** 10.1038/srep46873

**Published:** 2017-06-30

**Authors:** Frederik Holst, Erling A. Hoivik, William J. Gibson, Amaro Taylor-Weiner, Steven E. Schumacher, Yan W. Asmann, Patrick Grossmann, Jone Trovik, Brian M. Necela, E. Aubrey Thompson, Matthew Meyerson, Rameen Beroukhim, Helga B. Salvesen, Andrew D. Cherniack

Scientific Reports 6: Article number: 2552110.1038/srep25521; published online: 05 10 2016; updated: 06 30 2017

This Article contains errors in the frequencies of *ESR1* amplifications concerning uterine corpus endometrioid carcinoma (UCEC) in TCGA, which were incorrectly given as 88 cases (16.3%) and 36 cases (6.7%) for overall and focal amplifications respectively. These numbers represent the regarding amplification frequencies of partial *ESR1* sequences that manifest the GISTIC peak.

The correct frequencies of *ESR1* amplification in UCEC of TCGA are 90 cases (16.7%) and 39 cases (7.2%) for overall and focal amplification respectively. Accordingly, the seven cases with *ESR1* amplifications that appear to truncate the hormone-binding domain encoding region manifest 17.9% instead of 19.4% of cases with focal amplification.

In addition, the GISTIC q-value for *ESR1* amplification is incorrectly given as “q = 5.75 × 10^−4^”. The correct term and number for this value is “residual q = 2.29 × 10^−4^ after ‘GISTIC peel-off’”.

As a result, in the Results section under subheading ‘Hormone-binding domain truncated *ESR1* amplifications in primary endometrial cancers’.

“In the TCGA data subset of 539 endometrial carcinomas analyzed, we identified 88 (16.3%) cases with amplifications encompassing or overlapping *ESR1*. 46.6% of these were histologically defined serous and 75.0% of the tumors with *ESR1* amplification were clustered within the serous like copy-number high molecular subtype according to TCGA. The *ESR1* amplifications were focal (less than half a chromosome arm in length) in 36 cases (6.7%) of tumors, and had a significantly higher rate of amplification than the genome-wide average (q = 5.75 × 10^−4^). Mapping of the overlap between amplifications across tumors identified *ESR1* only as the most likely gene target (see methods).

These amplifications appeared to truncate the hormone-binding domain encoding region in seven cases (1.3% of the entire dataset; and 19.4% of cases with focal *ESR1* amplification) and to retain exons 1–4 or 1–3, encoding the n-terminal *ESR1* transactivation domain (AF1) and DNA-binding domains. Another case without *ESR1* amplification exhibited a heterozygous deletion of exons encoding the hormone-binding domain (Fig. 2), for a total apparent *ESR1* truncation rate of 1.5% over all tumors. In one additional TCGA case, we detected a hormone-binding domain (exons 4–8) truncating *ESR1-SYNE1* mRNA fusion (Appendix B). Eight of these nine tumors were molecularly classified as being in the serous like copy-number high subgroup (4.3% of this subgroup)”.

should read:

“In the TCGA data subset of 539 endometrial carcinomas analyzed, we identified 90 (16.7%) cases with amplifications encompassing or overlapping *ESR1*. 45.6% of these were histologically defined serous and 75.0% of the tumors with *ESR1* amplification were clustered within the serous like copy-number high molecular subtype according to TCGA. The *ESR1* amplifications were focal (less than half a chromosome arm in length) in 39 cases (7.2%), and had a significantly higher rate of amplification than the genome-wide average (residual q = 2.29 × 10^−4^ after “GISTIC peel-off”). Mapping of the overlap between amplifications across tumors identified *ESR1* only as the most likely gene target (see methods).

These amplifications appeared to truncate the hormone-binding domain encoding region in seven cases (1.3% of the entire dataset; and 17.9% of cases with focal *ESR1* amplification) and to retain exons 1–4 or 1–3, encoding the n-terminal *ESR1* transactivation domain (AF1) and DNA-binding domains. Another case without *ESR1* amplification exhibited a heterozygous deletion of exons encoding the hormone-binding domain (Fig. 2), for a total apparent *ESR1* truncation rate of 1.5% over all tumors. In one additional TCGA case, we detected a hormone-binding domain (exons 4–8) truncating *ESR1-SYNE1* mRNA fusion (Appendix B). Eight of these nine tumors were molecularly classified as being in the serous like copy-number high subgroup (4.3% of this subgroup)”.

