# Supplementary material for: Recurrent hormone-binding domain truncated ESR1 amplifications in primary endometrial cancers suggest their implication in hormone independent growth
Source: Sci Rep. 2016 May 10;6:25521. doi: 10.1038/srep25521 (PMC4861919; doi:10.1038/srep25521)
Supplement: Supplementary Information [file srep25521-s1.pdf]

Recurrent hormone-binding domain truncated *ESR1* amplifications in primary endometrial cancers suggest their implication in hormone independent growth.

Frederik Holst, Erling A. Hoivik, William J. Gibson, Amaro Taylor-Weiner, Steven E. Schumacher, Yan W. Asmann, Patrick Grossmann, Jone Trovik, Brian M. Necela, E. Aubrey Thompson, Matthew Meyerson, Rameen Beroukhim, Helga B. Salvesen & Andrew D. Cherniack

Supplementary Figure S1

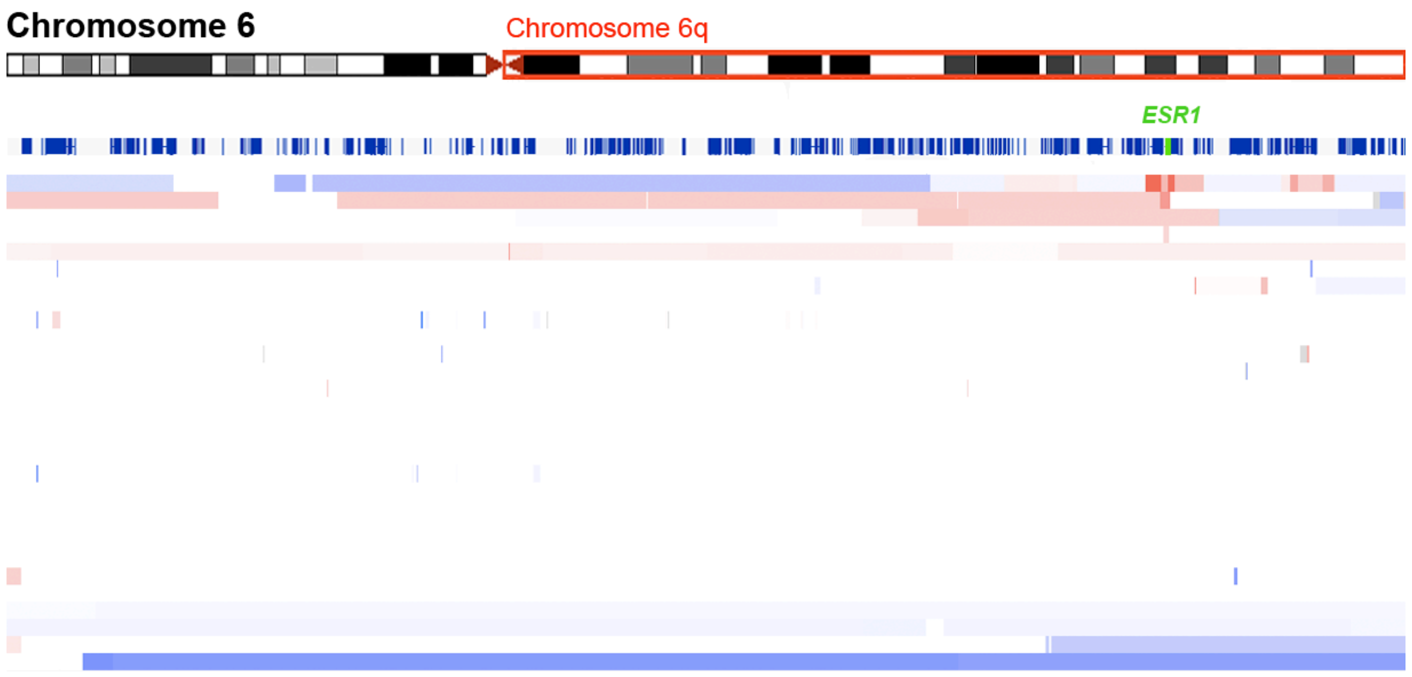

Supplementary Figure S1. Heatmap of copy number alterations on chromosome 6q in 29 primary endometrial carcinomas. Segmented log2 GeneChip copy number ratios of 29 metastatic high grade primary endometrial tumors on chromosome 6q are shown in horizontal bars (red: increased, white: neutral / normal, blue: decreased / deleted). Sort order and copy number data of tumors are according to segmented log2 values in Appendix A. Positions of genes are indicated in dark blue. Position of *ESR1* is highlighted green.

Recurrent hormone-binding domain truncated *ESR1* amplifications in primary endometrial cancers suggest their implication in hormone independent growth.

Frederik Holst, Erling A. Hoivik, William J. Gibson, Amaro Taylor-Weiner, Steven E. Schumacher, Yan W. Asmann, Patrick Grossmann, Jone Trovik, Brian M. Necela, E. Aubrey Thompson, Matthew Meyerson, Rameen Beroukhim, Helga B. Salvesen & Andrew D. Cherniack

Supplementary Figure S2

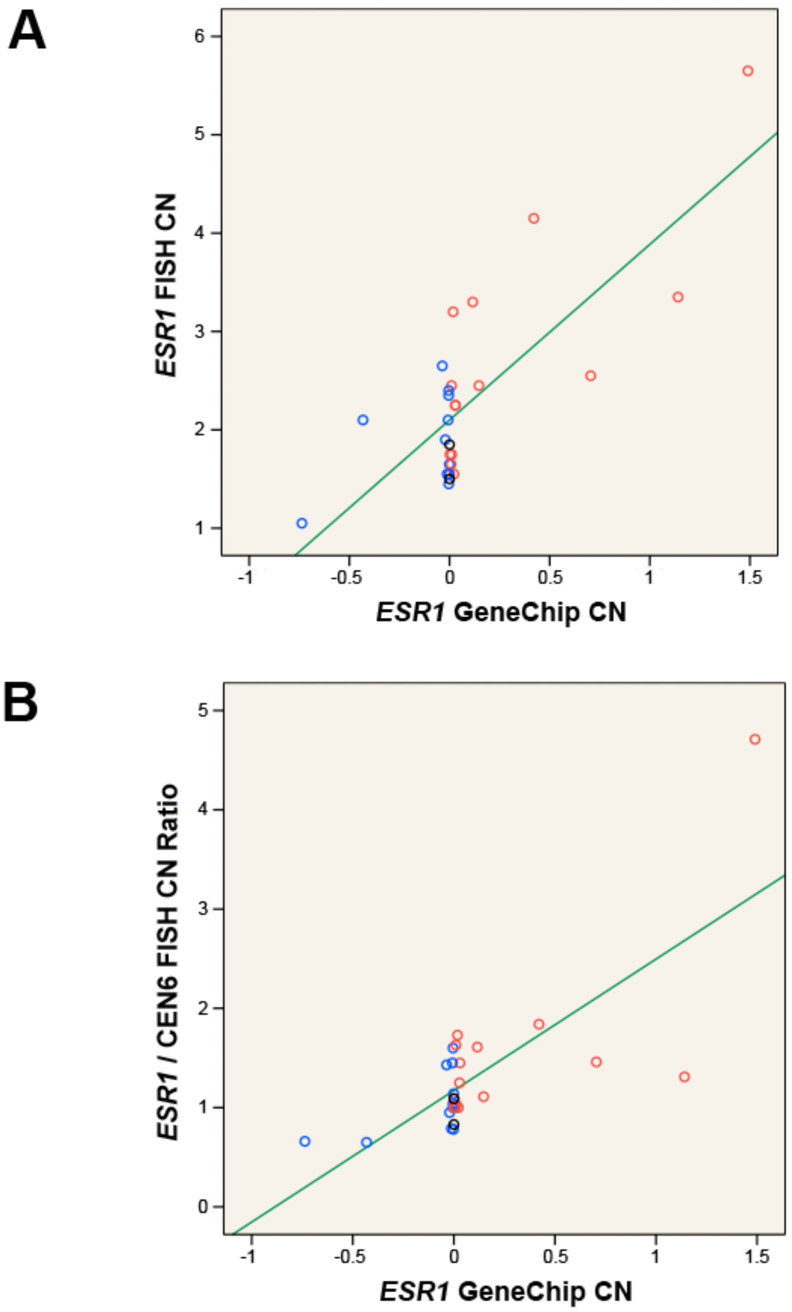

Supplementary Figure S2. Variance of GeneChip against FISH copy number in 28 out of 29 primary endometrial carcinomas. Scatter plots show the linear relative copy number from baseline ploidy determined by Affymetrix SNP 6.0 GeneChips (x-axis) in relation to the absolute average *ESR1* copy numbers per nucleus **(A)** and average *ESR1* to centromere 6 (CEN6) ratios **(B)** determined by FISH (y-axis) from a consecutive subset of 28 metastatic high grade primary endometrial tumors. GeneChip values > and <0.00 are highlighted red and blue respectively. Fit lines at total are shown in green.

Recurrent hormone-binding domain truncated *ESR1* amplifications in primary endometrial cancers suggest their implication in hormone independent growth.

Frederik Holst, Erling A. Hoivik, William J. Gibson, Amaro Taylor-Weiner, Steven E. Schumacher, Yan W. Asmann, Patrick Grossmann, Jone Trovik, Brian M. Necela, E. Aubrey Thompson, Matthew Meyerson, Rameen Beroukhim, Helga B. Salvesen & Andrew D. Cherniack

Supplementary Optical Dataset S1:

http://dx.doi.org/10.6084/m9.figshare.1517577

Dataset description

Optical dataset illustrating *ESR1* FISH signal appearance by full size FISH photographs of a total of 28 out of 29 primary endometrial cancers that had gone on to metastasize and that had copy-number changes characterized by SNP GeneChips (tumors #1-17 and #19-29).
The three color photographs show the nuclear *ESR1* FISH signals in 4µm conventional large section FFPE tissue slides. Shown are *ESR1* signals (green), CEN6 signals (orange) and nuclei (blue).
Photographs were taken using an Olympus IX51 microscope at 100× magnification, an Olympus XM10 digital camera (1,376 × 1,032 pixels) and Olympus cellSens imaging software.
Increased *ESR1* FISH signals are marked exemplarily in some cases (white arrows and edges). These could reach from two signals nearby each other, for gene duplication to several green dots.
For all 28 tumors pictures of up to four different tissue areas (Fig. 01-04) are available, including up to four different z-layers (Z-Stack A-D) per area to illustrate the distribution of signals within the z-axis.
